# Hand-to-face contact behaviors during indoor activities in daily life among Korean adults: an observational pilot study using videotaping

**DOI:** 10.4178/epih.e2021030

**Published:** 2021-04-22

**Authors:** Hyang Soon Oh, Mikyung Ryu, Youngran Yang

**Affiliations:** 1 Department of Nursing, College of Life Science and Natural Resources, Sunchon National University, Suncheon, Korea; 2 Department of Nursing, College of Nursing and Public Health, Daegu University, Daegu, Korea; 3 Research Institute of Nursing Science, Jeonbuk National University College of Nursing, Jeonju, Korea

**Keywords:** Activities of daily living, Cross infection, Disease transmission, Hand hygiene, Contact tracing, Touch

## Abstract

**OBJECTIVES:**

Hand-to-face contact (HFC) is the major route for the self-inoculation of pathogens. This study aimed to describe the characteristics of HFC behaviors among Korean adults during indoor activities.

**METHODS:**

Thirty participants were enrolled in the study, and 2 hours of videotaped data were collected from each participant. Contact data were recorded by examining the frequency and duration of HFC on the videos. Three training sessions were conducted for 2 readers to ensure the accuracy and reliability of videotape reading. Re-reading and verification of selected video data were performed to confirm intrapersonal and interpersonal validity. Contact exposure (CE) was determined by multiplying the contact frequency (CF) by the contact duration (CD) to quantify the intensity of contact during the observation time (2 hours).

**RESULTS:**

A total of 3,007 HFCs (1,305 mucous membrane contacts and 1,702 non-mucous membrane contacts) were observed for 60 person-hours. The median CF (person/2 hr) of the mucous membranes (eye; 4.0, nose; 15.5, mouth; 16.5) was 39.5/person and the median total CD was 177.0 sec/person. The median CE (frequency-duration/sec/person) was 5,795.0 (mouth: 1,356.0, nose: 600.0, eye: 57.5).

**CONCLUSIONS:**

This study showed that the mouth and nose were the most frequent exposure sites for HFC. Avoiding habitual HFC, awareness of self-inoculation by HFC, and vigorous hand hygiene should be strengthened to prevent the spread of infections.

## INTRODUCTION

Many kinds of infectious diseases, including recent 21st-century diseases such as severe acute respiratory syndrome (SARS) and Middle Eastern respiratory syndrome (MERS), have threatened public health despite meaningful developments in infection prevention techniques. The most frequent mode of transmission of such infectious diseases is contact with contaminated hands; therefore, strict hand hygiene and avoidance of hand-to-face contact (HFC) are strongly recommended to prevent the transmission of infections [1,2].

HFC is the major route for self-inoculation of pathogens [3]. When someone touches his or her eyes, nose, or mouth with contaminated hands, self-inoculation of pathogens can occur. Self-inoculation is a type of hand contact transmission where a person’s contaminated hands make subsequent contact with other sites on the person’s body, introducing contaminated material [4]. Self-inoculation via HFC in individuals leads to further opportunities for transmission; thus, it can constitute a potential transmission route of infectious diseases in the community [4,5]. This behavior can be particularly relevant during pandemics, such as SARS, MERS, and novel influenza A (H1N1) [6].

Studies on self-inoculation via HFC have been conducted for respiratory infections [5,7] and *Staphylococcus aureus* infections [8] among healthcare workers [9] and biosafety workers [10]. A substantial proportion of human respiratory tract infections are thought to be transmitted via contact between contaminated hands and the mucous membranes of the mouth, eyes, and/or nose, and a key risk factor for infection transmission is the rate of hand contact with these areas, termed “target facial mucous membranes” [5]. Respiratory viral tract infections such as rhinovirus [7,11] and H1N1 [6] have been reported to be transmitted by self-inoculation. One study reported an HFC rate per hour of 15.7 and proposed an equation to predict the transmission of respiratory diseases [5].

Therefore, understanding the characteristics of HFC is important for estimating infectious pathogen exposure in addition to chemical exposure [12]. However, few studies have described the frequency or patterns of HFC in terms of infectious exposures, despite strong recommendations to avoid HFC [13]. Studies on HFC have mostly focused on chemical exposures [12,14]. Additionally, the reported characteristics of HFCs in previous studies have varied [4,9,10,12,14,15].

Moreover, studies on the patterns and frequencies of HFC in the Korea have not been conducted yet, as a means of self-inoculation of infectious pathogens in Korea. Therefore, evidence on ways to avoid HFC as a means of infection prevention is scarce. This study conducted a brief survey to describe the frequency, duration, and intensity of HFC among Korean adults during daily indoor activities as a pilot study to provide evidence for estimating pathogen exposure according to HFC behavior.

## MATERIALS AND METHODS

### Study setting and participants

From January 14, 2018 to February 12, 2018, participants were selected by convenience sampling. A total of 30 Korean adults aged ≥ 20 years participated, including 10 university students from one university, 10 university students, faculty members, and faculty assistants from another university, and 10 participants from a church congregation. We first explained the purpose and method of this study to the directors of the institutions. We then explained the requirements for videotaping indoor activity data during daily life to the participants. Participants who voluntarily agreed to take part in this study were enrolled and their consent was obtained for videotaping their activities. The participants understood and agreed that the researchers would measure their behaviors from the recorded tapes, which would be kept confidential to protect their privacy. To reduce the possibility of consciously limiting HFC behavior by the participants during the videotaping, we did not precisely inform them about the specific HFC behaviors being observed for the purposes of the study. After recording their behaviors by videotaping, participants were then advised of the precise aspects of HFC shown on their behavior recordings. They subsequently consented to our measurement of HFC after the video recordings. The videotaping locations were chosen to facilitate indoor activities in a daily living environment, such as a lecture room for university students, workplace offices for faculty members and faculty assistants, and a church chapel for church congregation members. Participants’ behaviors were recorded via videotape for 2 hours each (60 person-hours) and were measured during the most active time on a day that was convenient to participants. Participants performed their daily routine tasks involving indoor activities (such as lectures, discussions, reading, writing, computer work, worshipping, or Bible study) as usual during the videotaping.

### Data collection/procedure

Data on the contact frequency (CF) of HFCs, contact duration (CD) of HFCs, and HFC areas were recorded for each participant in a standardized Excel form. The reading time to check each CD was recorded in the form of start minute, end minute, start second, and end second (referring to the timestamp at the bottom of the video recording) to avoid missing time. If the reading time was missed, the video playback was checked to prevent data loss. Contact areas were observed, and HFC was classified in terms of whether it involved contact with mucous membranes.

### Data accuracy and reliability

To ensure the accuracy and reliability of the video reading, we secured 2 video readers to enable cross-verification, and we conducted 3 training sessions. To reduce intrapersonal reading errors, 4% of the data entered by each reader was re-read and checked for contact areas, CF, and CD to ensure a match of more than 90%. The minimization of interpersonal reading errors was confirmed by an agreement of more than 90% in approximately 10% of randomly selected readings taken by the other reader.

### Definition of terms

Mucous membrane contacts were defined as HFCs involving the eye, nose, and mouth, and non-mucous membrane contacts were defined as HFCs involving the skin of the head, forehead, chin, cheek, and ear.

We defined the contact exposure (CE) index by multiplying CF and CD to estimate HFC exposure. CE was defined to quantify the total number of HFCs during 2 hours of observation.

### Statistical analysis

Continuous variables and categorical variables were presented as mean ± standard deviation or median (25th percentile; 75th percentile), interquartile range, and frequencies and percentages, respectively. We used the Kruskal-Wallis test and the Fisher exact test to compare the demographic characteristics of the 3 groups recorded in different settings (chapel, classroom, and office) according to the data type. The Dunn post-test and Kruskal-Wallis test were used for multiple comparisons to investigate the differences in contact indices between chapel, classroom, and office settings. Statistical significance was set at p < 0.05. Data analyses were performed using R 3.3.3 (version 3.15) for Windows (R Foundation for Statistical Computing, Vienna, Austria).

### Ethics statement

All procedures performed in this study involving human participants followed the ethical standards of the appropriate institutional research committee (IRB No. 1040173–201712-HR-033-02) and were conducted according to the 1964 Helsinki Declaration and its later amendments or comparable ethical standards. Informed written consent was obtained from all individual participants in this study before video recording and after video recording.

## RESULTS

### General characteristics of participants

The average age of the 30 participants was 41.0 years. Those in their 20s accounted for 43.3%; 63.3% were female, 51.7% were high school graduates, and 27.6% were university students (Table 1). Table 1 shows the general characteristics of the participants in each indoor setting (chapel, classroom, and office).

### Descriptive statistics of self-contact and hand-to-face contact

The descriptive statistics of HFC are shown in Table 2. A total of 30 participants were observed, making 3,007 HFCs during 2 hours of observation. Of the HFCs, 43.4% (1,305 contacts) involved contact with mucous membranes such as the eyes, nose, and mouth.

The median frequency (n/person) of HFCs per person was highest for the mouth (16.5), followed by the nose (15.5) and head (13.0). The median CD (sec/person) of mucous membrane HFCs per person was longest for the head (111.5), followed in order by the chin (85.0), mouth (72.5), cheek (51.5), and nose (42.5). The median CD per contact (sec/contact) of mucous membrane HFCs was the longest for the mouth (4.5), followed by the eye (4.1) and nose (3.4). The median CD per contact (sec/contact) of non-mucous membrane HFCs was the longest for the chin (13.2), followed by the cheek (6.0) and head (5.7). The mean values of CF, CD, and CE are also presented in Table 2, and the rankings of frequency according to mean values were consistent with those obtained using median values (Table 2).

**Figure 1.**
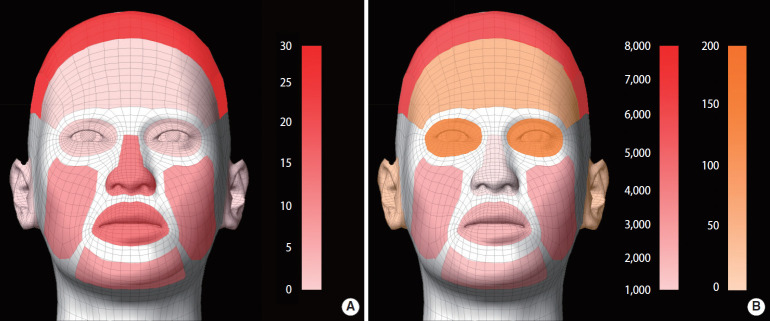
Visualization of the frequency and contact exposure of hand-to-face contact. (A) Visualization of the frequency of hand-to-face contact. The color bar expresses the contact frequency on a scale from 0 to 30; the darker the color, the more frequent the contact. (B) Visualization of hand contact exposure to the face. The more contact exposure (frequency-duration/sec/person), the darker the color, ranging from orange to red.

The median CE (/sec/person) for contacts involving mucous membranes was the longest for the mouth (1,356.0), followed by the nose (600.0) and eye (57.5). The median CE for non-mucous membrane contacts was the longest for the head (1,415.0), followed by the chin (523.5) and cheek (331.0). Figure 1 visualizes the HFC frequency and exposure.

### Differences in contact indices of hand-to-face contact by the setting of indoor activities

Table 3 shows that there was no significant difference in the median CF of the mucous membrane (eye, nose, and mouth) HFCs according to different indoor settings. The median CF of non-mucous membrane HFCs was different for head contact (higher in the classroom setting) (p<0.001). The median CD for mucous membrane contact with the mouth was significantly higher among the participants in classroom settings (p=0.026). The median CE was significantly higher for the mouth (p=0.037) and head (p<0.001) among the participants in the classroom setting.

## DISCUSSION

This pilot study conducted to describe the frequency, duration, and intensity of HFC among Korean adults is the first study of HFC conducted in Korea. The results of this study improve our understanding of HFC patterns among Korean adults during indoor activities in daily life and provide evidence for estimating exposure to pathogens via HFC, thereby furnishing strong support for hand hygiene and the need to avoid HFC.

Participants’ age varied widely because they were selected from adults ≥ 20 years of age to include age groups broadly representative of the adult population.

In this study, almost half of the HFCs involved contact with mucous membranes (eye, nose, and mouth). This finding is consistent with a previous study by Kwok et al. [4] who reported a proportion of mucosal contact of 44.0% and non-mucosal contact of 56.0%.

The average CF of HFC in this study was higher than those reported in previous studies: 23/person/hr [4], and 15.7/person/hr [5], 9.5/person/hr [9], and 2.6/person/hr [10]; however, our results are consistent with the finding of 27.7 (6-49)/45 min of another study [12]. The most frequently encountered non-mucous membrane and mucous membrane HFC areas in this study were the head and the mouth, nose, and eyes, respectively. This is consistent with a previous study (head and mouth: 4/hr, nose: 3/hr, and eyes: 3/hr) [4], the head was reported as the most frequent contact area [14], and mouth contacts occurred twice as often as nose or eye contacts [9]. However, this finding is inconsistent with results reporting the nose as the highest contact area (44.9%) in biosafety level-2 workers [10]. The most frequent HFC area of the face has been reported to vary according to nationality, with British people frequently touching the chin and mouth, while Japanese people frequently touched the nose and eyes [15]. Since HFC studies have reported various results in different study populations, further research will be needed to develop effective preventive measures regarding HFC for Koreans.

The average CD of HFCs (sec/contact) was highest for the head, followed in order by the mouth, nose, and eyes. This order corresponds to that of a previous report, but with longer durations [4]. Among mucous membrane contacts, the mouth was confirmed to have the highest CE index, followed by the nose.

In the comparative analysis of the 3 group settings, the indices of CF, CD, and CE were higher for the mouth and head in the college student group in classroom settings than other two groups in other settings, which is consistent with the characteristics of HFC observed in young adults [4,16]. Further research will be needed to identify differences in HFC according to age groups and different settings of indoor activities.

In this study, HFC was more frequent and longer than observed in previous studies [4,9,10]. The mouth showed the highest CF, CD, and CE of HFC, followed by the nose. The rate of HFC in this study is sufficient to predict respiratory disease acquisition according to Nicas & Best [5]. These results suggest that the mouth and nose may be major exposure routes of self-inoculation in Korean adults. In addition to the mouth [17,18], particular attention should be paid to the nasal mucosa as a source of respiratory infections [3], especially given its large number of resident microbes, and it is associated with a high risk of spreading infections to other parts of the nasal passages via the hands [19]. Therefore, this study’s results will be useful for reducing the risk of respiratory tract infections by informing efforts to reduce HFC to the nose [3,20]. Though not as risky as HFC to mucous membranes, non-mucous membrane HFC of the head and cheek should also be avoided because the face can become contaminated if touched with unwashed hands, and this may subsequently enable the spread of pathogens to mucous membranes via HFC [21].

The CF or CD indices were useful for comparing the findings of the present study with those of previous studies reporting CF or CD [4,5,9,10,14,15]. However, these indices alone have limitations in terms of estimating the total exposures via HFC. The CE index was more useful for differentiating and clearly distinguishing between mouth and nose exposures via HFC than the simpler indices of CF or CD of the mouth and nose alone, and therefore more useful for estimating the exposure risk via HFC.

In addition, the results of this study could be used as strong evidence for the importance of following good respiratory hygiene, which involves regularly and thoroughly cleaning one’s hands, avoiding touching one’s eyes, nose, and mouth with one’s hands, and covering one’s mouth and nose with a bent elbow or tissue when coughing or sneezing [22].

Education is needed to raise awareness of the risks of HFC, and strategies need to be developed to reduce habitual HFC behaviors [20,21,23]. The risk of infectious disease transmission through HFC can be prevented through the modification of HFC behaviors [23]. Furthermore, during a pandemic (such as the coronavirus disease 2019 [COVID-19] pandemic), the use of masks can be a part of a comprehensive package for prevention and control measures in different settings [24]. These precautions can protect the mouth and nose, which were identified as possible routes for infectious microbe inoculations via hand contact.

This study had some limitations. It measured HFCs during indoor activities within a limited time period with a limited number of participants, and was the first such study in Korea. Furthermore, it used convenience sampling. Therefore, it was not possible to analyze all HFCs over a 24-hour period or capture every situation, and therefore the generalizability of the findings to the entire Korean population is limited. Further studies will be required for higher generalizability. Nevertheless, this study described in detail the characteristics of HFC for the first time, and the findings can be used as effective evidence of self-inoculation via HFC and provide evidence for good hand hygiene and avoiding HFC. Furthermore, the results of this study will be useful for developing exposure models and control strategies to prevent infectious disease transmission via HFC, especially during pandemics such as COVID-19.

In conclusion, this study showed that the CF and CD of HFC were more frequent and longer than reported in previous studies. The most frequent HFC exposure area was the mouth, followed by the nose. Therefore, the mouth and nose may be the most frequent exposure routes for infectious pathogens. The CE index may be more useful than other indices for measuring total exposure via HFC. Avoiding habitual HFC to the mouth and nose, building awareness of self-inoculation via HFC, and following good respiratory hygiene should be promoted to prevent exposure to pathogens. Furthermore, studies on HFC in various situations, specific age groups, and larger populations will be needed to generalize the findings to the Korean population.

## Figures and Tables

**Table 1.** General characteristics of participants (n=30)

Characteristics		Total	Chapel (n=10)	Classroom (n=10)	Office (n=10)	p-value^1^
Age (yr)						<0.001
	20-29	13 (43.3)	0 (0.0)	10 (100.0)	3 (30.0)	
	30-59	10 (33.3)	4 (40.0)	0 (0.0)	6 (60.0)	
	≥60	7 (23.3)	6 (60.0)	0 (0.0)	1 (10.0)	
	Mean±SD (median)	41.0±18.5 (43.0)	60.0±9.6 (60.0)	21.4±1.5 (21.0)	41.6±13.3 (43.5)	<0.001
Sex						0.510
	Male	11 (36.7)	5 (50.0)	4 (40.0)	2 (20.0)	
	Female	19 (63.3)	5 (50.0)	6 (60.0)	8 (80.0)	
Marital status						<0.001
	Single	14 (46.7)	0 (0.0)	10 (100)	4 (40.0)	
	Married	16 (53.3)	10 (100)	0 (0.0)	6 (60.0)	
Education						<0.001
	High school and under	15 (51.7)	7 (77.8)	8 (80.0)	0 (0.0)	
	College (undergraduate)	8 (27.6)	2 (22.2)	2 (20.0)	4 (40.0)	
	Graduate school	6 (20.7)	0 (0.0)	0 (0.0)	6 (60.0)	
Occupation						<0.001
	None	5 (16.7)	5 (50.0)	0 (0.0)	0 (0.0)	
	Student	11 (36.7)	0 (0.0)	10 (100)	1 (10.0)	
	Officer	5 (16.7)	2 (20.0)	0 (0.0)	3 (30.0)	
	Sales/service	1 (3.3)	1 (10.0)	0 (0.0)	0 (0.0)	
	Health care provider	1 (3.3)	0 (0.0)	0 (0.0)	1 (10.0)	
	Others	7 (23.3)	2 (20.0)	0 (0.0)	5(50.0)	
Economic activity						<0.001
	Unemployed (including students)	16 (53.3)	5 (50.0)	10 (100)	1 (10.0)	
	Employed	14 (46.7)	5 (50.0)	0 (0.0)	9 (90.0)	
Household income/mo (104 KRW)						0.008
	<300	7 (25.0)	5 (62.5)	2 (20.0)	0 (0.0)	
	300-<500	8 (28.6)	3 (37.5)	3 (30.0)	2 (20.0)	
	500-<1,000	11 (39.3)	0 (0.0)	5 (50.0)	6 (60.0)	
	≥1,000	2 (7.1)	0 (0.0)	0 (0.0)	2 (20.0)	
Residence						0.012
	Metropolitan	18 (60.0)	8 (80.0)	8 (80.0)	2 (20.0)	
	City or small town	12 (40.0)	2 (20.0)	2 (20.0)	8 (80.0)	

Values are presented as number (%). SD, standard deviation; KRW, Korean won.

1 The Kruskal-Wallis test was used for continuous variables and the Fisher exact test for categorical variables.

**Table 2.** Descriptive statistics of contact indices (n=30, 2-hour observation time)

Variables			Observed contacts (n=3,007)	Mean±SD	1Q	Median	3Q	IQR
Contact frequency (n/person)^1^								
	Mucous membranes		1,305	43.5±19.4	33.5	39.5	51.5	18.0
		Eye	180	6.0±5.6	2.0	4.0	9.5	7.5
		Nose	541	18.0±13.8	9.3	15.5	19.5	10.3
		Mouth	584	19.5±16.2	8.0	16.5	27.0	19.0
	Non-mucous membranes		1,702	56.7±40.4	26.0	49.0	74.5	48.5
		Head	835	27.8±29.0	7.3	13.0	48.5	41.3
		Forehead	80	2.7±3.4	0.0	2.0	3.8	3.8
		Chin	326	10.9±12.6	3.3	6.0	14.8	11.5
		Cheek	372	12.4±17.0	3.3	6.5	14.0	10.8
		Ear	89	3.0±3.2	1.0	2.0	3.8	2.8
Contact duration (sec/person)^2^								
	Mucous membranes		1,305	232.9±227.4	111.5	177.0	256.5	145.0
		Eye	180	30.4±35.5	7.0	17.0	39.5	32.5
		Nose	541	73.3±61.6	30.0	42.5	99.8	69.8
		Mouth	584	129.3±215.0	19.0	72.5	124.8	105.8
	Non-mucous membranes		1,702	767.6±749.3	179.0	521.0	1,184.5	1,005.5
		Head	835	292.7±486.9	29.5	111.5	375.3	345.8
		Forehead	80	26.4±74.8	0.0	6.5	18.8	18.8
		Chin	326	195.6±323.4	28.0	85.0	160.5	132.5
		Cheek	372	224.8±434.3	17.3	51.5	119.8	102.5
		Ear	89	28.1±44.1	1.5	8.0	45.0	43.5
Contact duration per contact (sec/contact)^3^								
	Mucous membranes		1,305	5.0±3.0	2.9	3.9	6.0	3.1
		Eye	180	4.7±3.3	3.0	4.1	5.1	2.1
		Nose	541	4.4±3.7	2.4	3.4	4.5	2.1
		Mouth	584	5.9±3.5	2.4	4.5	6.5	4.0
	Non-mucous membranes		1,702	13.1±11.3	6.3	8.2	17.7	11.4
		Head	835	9.3±10.1	3.8	5.7	10.1	6.2
		Forehead	80	8.0±9.1	2.7	3.5	9.3	6.6
		Chin	326	13.6±8.3	8.5	13.2	17.7	9.2
		Cheek	372	17.3±43.1	4.2	6.0	9.6	5.4
		Ear	89	11.0±19.1	2.4	5.0	10.8	8.4
Contact exposure (frequency-duration/sec/person)^4^								
	Mucous membranes		1,305	12,920.5±18,978.9	4,087.0	5,795.0	12,553.5	8,466.5
		Eye	180	314.0±489.2	14.0	57.5	444.5	430.5
		Nose	541	1,833.4±3,204.0	312.8	600.0	2,361.8	2,049.0
		Mouth	584	5,309.6±14,691.7	128.0	1,356.0	3,360.5	3,232.5
	Non-mucous membranes		1,702	63,290.6±89,051.4	5,860.8	25,683.0	79,055.3	73,194.5
		Head	835	17,433.8±45,438.6	222.0	1,415.0	14,162.3	13,940.3
		Forehead	80	231.1±897.9	0.0	13.0	72.0	72.0
		Chin	326	5,824.4±17,158.5	120.0	523.5	1,845.8	1,725.8
		Cheek	372	6,903.3±21,456.1	40.5	331.0	1,978.5	1,938.0
		Ear	89	157.1±302.4	1.5	18.0	134.3	132.8

SD, standard deviation; 1Q, 25th percentile; 3Q, 75th percentile; IQR, interquartile range.

1 Contact frequency is the average number of hand-to-face contacts per person for 2-hours observation.

2 Contact duration is the average second of hand-to-face contacts per person for 2-hours observation.

3 Contact duration per contact=^2^/^1^.

4 Contact exposure (^1^x^2^) was defined by multiplying the contact frequency by the contact duration.

**Table 3.** Differences in the contact indices of hand-to-face contact by the settings of indoor activities (n=30, 2-hour observation time)

Variables			Indoor activity settings			p-value (post-hoc)^4^
			Chapel (n=10)^1^	Classroom (n=10)^2^	Office (n=10)^3^	
Contact frequency (n/person)^5^						
	Mucous membranes		39.5 [33.0; 45.0]	54.5 [39.0; 79.0]	35.5 [22.0; 49.0]	0.148
		Eye	3.0 [2.0; 10.0]	3.5 [3.0; 6.0]	5.0 [2.0; 12.0]	0.624
		Nose	10.5 [8.0; 18.0]	13.0 [10.0; 18.0]	16.0 [15.0; 21.0]	0.307
		Mouth	16.5 [12.0; 27.0]	20.5 [14.0; 48.0]	6.0 [2.0; 18.0]	0.054
	Non-mucous membranes		25.0 [12.0; 32.0]	73.0 [63.0; 106.0]	53.0 [26.0; 103.0]	0.001 (2,3>1)
		Head	5.5 [4.0; 10.0]	50.5 [17.0; 62.0]	11.5 [8.0; 35.0]	0.001 (2,3>1)
		Forehead	0.0 [0.0; 2.0]	2.0 [0.0; 4.0]	3.0 [1.0; 8.0]	0.146
		Chin	3.0 [2.0; 6.0]	7.0 [5.0; 27.0]	7.0 [5.0; 17.0]	0.067
		Cheek	6.5 [3.0; 14.0]	5.5 [3.0; 10.0]	13.0 [4.0; 22.0]	0.332
		Ear	1.5 [0.0; 2.0]	3.0 [1.0; 8.0]	2.5 [1.0; 4.0]	0.189
Contact duration (sec/person)^6^						
	Mucous membranes		119.0 [100.0; 188.0]	267.5 [184.0; 324.0]	152.0 [110.0; 237.0]	0.076
		Eye	16.5 [5.0; 74.0]	18.0 [12.0; 35.0]	20.5 [6.0; 41.0]	0.961
		Nose	40.5 [30.0; 66.0]	36.5 [28.0; 104.0]	100.0 [34.0; 147.0]	0.239
		Mouth	48.5 [18.0; 110.0]	123.5 [92.0; 224.0]	23.5 [7.0; 70.0]	0.026 (2>1,3)
	Non-mucous membranes		175.0 [92.0; 201.0]	1,182.0 [617.0; 2091.0]	587.0 [407.0; 1362.0]	0.001 (2,3>1)
		Head	26.0 [15.0; 66.0]	424.0 [210.0; 599.0]	88.0 [37.0; 381.0]	<0.001 (2>1,3)
		Forehead	0.0 [0.0; 7.0]	9.0 [0.0; 22.0]	18.5 [2.0; 70.0]	0.034 (3>1,2)
		Chin	26.0 [8.0; 111.0]	114.5 [59.0; 391.0]	80.5 [57.0; 226.0]	0.153
		Cheek	44.5 [17.0; 110.0]	46.0 [18.0; 59.0]	94.0 [9.0; 466.0]	0.780
		Ear	3.0 [0.0; 11.0]	35.5 [9.0; 74.0]	6.0 [1.0; 36.0]	0.053
Contact duration per contact (sec/contact)^7^						
	Mucous membranes		3.6 [2.5; 4.7]	4.8 [3.8; 6.6]	3.5 [2.8; 7.7]	0.188
		Eye	5.0 [4.2; 5.7]	4.7 [3.6; 4.8]	3.1 [2.5; 4.0]	0.096
		Nose	3.5 [2.3; 4.6]	3.4 [2.6; 3.9]	3.3 [2.3; 9.2]	0.830
		Mouth	2.9 [2.1; 4.5]	5.4 [4.8; 9.0]	4.4 [2.3; 6.2]	0.064
		continued				
	Non-mucous membranes		6.2 [5.5; 6.7]	14.9 [7.3; 25.4]	11.4 [7.2; 17.6]	0.020 (2,1>3)
		Head	5.3 [3.5; 5.7]	9.6 [5.7; 21.1]	5.9 [2.8; 12.3]	0.054
		Forehead	2.7 [1.7; 3.4]	3.5 [ 2.7; 7.7]	6.2 [3.7; 20.7]	0.094
		Chin	8.7 [4.0; 13.3]	16.2 [12.6; 21.0]	13.2 [11.2; 14.5]	0.141
		Cheek	6.4 [3.5; 8.5]	6.0 [5.0; 9.6]	5.5 [3.0; 19.3]	0.627
		Ear	3.2 [2.2; 3.7]	9.2 [7.2; 13.8]	2.4 [2.0; 5.6]	0.025 (2>1,3)
Contact exposure (frequency-duration/sec/person)^8^						
	Mucous membranes		5,191.5 [4,000.0; 6,642.0]	13,497.0 [6,560.0; 29,160.0]	5,153.0 [3,960.0; 12,462.0]	0.076
		Eye	49.5 [10.0; 740.0]	63.5 [36.0; 252.0]	107.0 [12.0; 492.0]	0.827
		Nose	427.5 [270.0; 912.0]	450.0 [240.0; 1872.0]	2,358.5 [544.0; 2,820.0]	0.113
		Mouth	756.0 [252.0; 2,025.0]	2,059.0 [1,656.0; 9,216.0]	132.5 [8.0; 1,260.0]	0.037 (2>1,3)
	Non-mucous membranes		5,169.5 [1,104.0; 6,432.0]	83,483.5 [43,190.0; 115,346.0]	39,077.0 [12,168.0; 77,634.0]	<0.001 (2>3>1)
		Head	172.0 [55.0; 738.0]	16,496.0 [7,849.0; 26,950.0]	855.5 [318.0; 14,946.0]	<0.001 (2>1,3)
		Forehead	0.0 [0.0; 14.0]	28.0 [0.0; 60.0]	53.0 [2.0; 256.0]	0.065
		Chin	72.0 [12.0; 1020.0]	766.5 [236.0; 12,121.0]	693.0 [330.0; 4,294.0]	0.148
		Cheek	331.0 [34.0; 1,540.0]	252.0 [54.0; 605.0]	1692.0 [36.0; 8,388.0]	0.506
		Ear	4.5 [0.0; 33.0]	103.5 [9.0; 592.0]	18.0 [1.0; 96.0]	0.084

Values are presented as median [25th percentile; 75th percentile].

1 Indoor activities in chapel congregational worship.

2 Indoor extracurricular activities in the classroom in a university.

3 Indoor administrative computer work, lecture, discussion, and writing activities in the offices in a university.

4 The Kruskal-Wallis test and post hoc test were pairwise comparisons using the Dunn test for multiple comparisons of independent samples using the Bonferroni adjustment method for p-values.

5 Contact frequency is the average number of hand-to-face contacts per person for 2-hours observation.

6 Contact duration is the average second of hand-to-face contacts per person for 2-hours observation.

7 Contact duration per contact=^6^/^5^.

8 Contact exposure (^5^x^6^) was defined by multiplying the contact frequency by the contact duration.
